# Construction and subgroup validation of a predictive model for IVIG-resistance in kawasaki disease

**DOI:** 10.3389/fcvm.2026.1808379

**Published:** 2026-08-13

**Authors:** Yuewen Li, Chuxiong Gong, Chunlan Zhang, Yi Huang, Houxi Bai, Jieqin Song, Houyu Chen, Xiaotao Yang, Xiaomei Liu

**Affiliations:** 1 Department of Clinical Immunology, Kunming Children’s Hospital & Children’s Hospital Affiliated to Kunming Medical University, Kunming, Yunnan, China; 2 Department of Cardiology, Kunming Children’s Hospital & Children’s Hospital Affiliated to Kunming Medical University, Kunming, Yunnan, China; 3 Strategic Operations Department, Kunming Children’s Hospital & Children’s Hospital Affiliated to Kunming Medical University, Kunming, Yunnan, China; 4 Kunming Maternal and Child Health Hospital, Kunming, Yunnan, China

**Keywords:** cardiovascular diseases, children's diseases, IVIG resistance, kawasaki disease, predictive model

## Abstract

**Objective:**

Kawasaki disease is an acute immune vasculitis, and intravenous immunoglobulin (IVIG) therapy was an important treatment method, although a small proportion of children exhibited IVIG resistance. Our study aimed to construct a risk model for Kawasaki disease with IVIG resistance and to validate it across different clinical characteristic subgroups, with the goal of optimizing personalized and precise management to improve patient outcomes.

**Methods:**

We first compared various factors between the groups with and without IVIG resistance. Then, we used LASSO analysis to screen for factors that significantly predicted outcomes. The selected factors were used to develop the risk model. We evaluated the model using ROC curves, calibration curves, and decision curve analysis, and conducted internal validation through 5-fold cross-validation. Finally, we performed subgroup analyses based on age, sex, and other factors.

**Results:**

Through univariate analysis, LASSO analysis, and correlation analysis, we identified WBC, PLT, CRP, fever days, PLR, and ALT as key factors in constructing the risk model. The model achieved an area under the curve of 0.816 (95% CI: 0.775–0.857). Additionally, calibration curves, DCA, and 5-fold cross-validation demonstrated that the model had good predictive performance. The model's effectiveness was also satisfactory across various subgroups.

**Conclusions:**

Our study successfully constructed a risk model for Kawasaki disease with IVIG resistance in the Chinese population. The model demonstrated strong predictive ability and was validated across multiple subgroups, showing potential clinical application value.

## 1 Introduction

Kawasaki disease (KD) is a systemic, non-specific acute vasculitis that primarily affects children under five years old (1). The disease is especially prevalent in East Asia, particularly in Japan, China, and South Korea (2). Epidemiological data indicated that the incidence in boys was approximately 1.5 times higher than in girls. The main treatment during the acute phase involved intravenous immunoglobulin (IVIG) combined with aspirin. Early administration of IVIG was proven to alleviate all clinical manifestations and significantly reduce the risk of coronary artery lesions (CAL) (3). However, some patients still experienced persistent or recurrent fever despite treatment, which was identified as IVIG resistance. Studies demonstrated that IVIG resistance increased the likelihood of developing CAL. Prompt identification and subsequent management of resistant cases helped mitigate this risk.

The mechanisms underlying IVIG resistance remained incompletely understood. Clinicians often used a second dose of IVIG or immunosuppressive agents such as corticosteroids and infliximab to manage resistance (4–6). Research revealed that younger children were more prone to experience IVIG resistance. Furthermore, certain clinical features and laboratory indicators, such as prolonged disease course, persistent high fever, elevated white blood cell count, and significantly increased ESR and CRP levels were associated with heightened inflammatory responses, disease severity, and IVIG resistance (7–9).

As a systemic inflammatory disease, KD exhibited notable heterogeneity due to individual differences, which influenced treatment outcomes. Factors such as age, gender, timing of treatment, and kinds of therapeutic agents affected the prognosis. Our prior studies employed principal component analysis (PCA) to uncover the existence of multiple subgroups within KD, which showed distinct differences in IVIG treatment outcomes (10). Based on these findings, our current research aimed to develop a predictive model for IVIG resistance, and to validate it across different clinical subgroups. This approach not only facilitated the identification of specific clinical characteristics and predictive efficacy within patient populations but also provided a foundation for personalized treatment strategies. A deeper understanding of the differences among these subgroups was expected to optimize KD management and improve patient prognoses.

## 2 Materials and methods

### 2.1 Participants

Our study retrospectively collected clinical data from 1,795 cases of Kawasaki disease diagnosed at Kunming Children's Hospital between December 2014 and December 2023. These cases represent an expansion based on previous research. All cases met the criteria for Kawasaki disease established by the American Heart Association (AHA). The study was conducted in accordance with the principles of the Declaration of Helsinki and received approval from the Ethics Committee of Kunming Children's Hospital (Ethics Approval No.: 2023-05-016-K01).

### 2.2 Diagnostic and inclusion/exclusion criteria

Diagnostic Criteria: All cases in our study conformed to the diagnostic standards for Kawasaki disease established by the American Heart Association (AHA), including complete Kawasaki disease (CKD) and incomplete Kawasaki disease (IKD) (1). CAL were defined as a maximum Z-score of the coronary arteries greater than 2.5 within 60 days of onset. Echocardiographic evaluations were performed by experienced pediatric cardiologists with over 10 years of specialization in pediatric echocardiography. The assessments were conducted at standardized intervals: during the acute phase (before IVIG), at 1–2 weeks, and at 4–8 weeks after disease onset. The evaluation of CAA was not based merely on routine clinical judgment but followed a structured protocol using Z-scores based on age, height, and weight parameters.

We utilized the coronary artery Z-score formulas that incorporate age, height, and weight (Body Surface Area, BSA), which are widely accepted and validated for the Chinese pediatric population. The specific equations used for the Left Main Coronary Artery (LMCA), Left Anterior Descending (LAD), and proximal Right Coronary Artery (pRCA) are based on the general formula: Z = [M - y]/MSE, where M represents the actual measured values to obtain the corresponding Z-scores.

Poor response to IVIG was defined as persistent fever above 38 °C after 36 h after completion of initial IVIG treatment, or recurrent fever within two weeks after initial treatment accompanied by at least one major clinical manifestation of Kawasaki disease, after excluding other potential causes of fever.

Inclusion Criteria: (1) Hospitalized children diagnosed with Kawasaki disease based on the Expert Consensus on the Diagnosis and Acute Treatment of Kawasaki Disease; (2) Availability of complete clinical data pertinent to Kawasaki disease patients. Exclusion Criteria: (1) Patients with pre-existing conditions such as cardiovascular diseases, liver diseases, kidney diseases, hematologic disorders, immune system disorders, or endocrine and metabolic genetic conditions; (2) Children with a history of previous Kawasaki disease; (3) Patients who received IVIG, aspirin, or glucocorticoids prior to hospital admission; (4) Patients with incomplete clinical data; (5) Presence of coronary artery lesions prior to IVIG treatment.

### 2.3 Inclusion factors

The data for our study were collected from the initial examinations conducted upon hospital admission prior to any treatment. The variables included in the analysis encompassed three demographic characteristics: age at onset, gender, and ethnicity. Laboratory data comprised following parameters: hemoglobin (Hb), white blood cell count (WBC), platelet count (PLT), neutrophil percentage (N), lymphocyte percentage (L), erythrocyte sedimentation rate (ESR), C-reactive protein (CRP), alanine aminotransferase (ALT), total bilirubin (TBIL), gamma-glutamyl transferase (GGT), albumin (ALB), globulin (GLB), bile acid levels, and levels of potassium (K), sodium (Na), chloride (Cl), magnesium (Mg), phosphorus (P), and calcium (Ca). Coagulation function tests included prothrombin time (PT), activated partial thromboplastin time (APTT), fibrinogen (Fbg), thrombin time (TT), and D-dimer levels. Immunoglobulin measurements involved IgA, IgM, and IgG. Physical examination findings recorded were oral mucosal involvement, conjunctival injection, cervical lymphadenopathy, limb symptoms, and rash. Additional data collected included the number of days with fever (Fever days), duration of fever before IVIG treatment (Time to IVIG treatment), and echocardiographic findings.

### 2.4 Model construction

Our study divided all cases into two groups based on resistance to IVIG treatment: IVIG-resistance group and non-IVIG-resistance group. We performed comparative analysis of clinical data between two groups. Variables with *P* < 0.05 were selected for further analysis. Next, we used the “glmnet” package in R to perform Least Absolute Shrinkage and Selection Operator (LASSO) analysis. This method identified key factors associated with prognosis. LASSO is a regularization technique that added an L1 penalty to the loss function. It selected variables by shrinking some coefficients to zero, simplifying the model. The main advantage of LASSO was its ability to retain only important variables. It reduced model complexity and prevented overfitting. We applied five-fold cross-validation to determine the optimal regularization parameter, *λ* (lambda.min and lambda.1se). Larger *λ* values resulted in stronger regularization and fewer variables. Selected variables were based on these *λ* values. To prevent multicollinearity, we conducted correlation analysis using the “corrplot” package. Then, we performed multivariate logistic regression with the “rmda” package. Key factors from this model were considered the final predictors. Finally, we plotted the nomogram of the best model using the “rms” package to visualize its predictive performance.

### 2.5 Model evaluation and validation

We used the “pROC” package in R to plot the receiver operating characteristic (ROC) curve and measured the model's performance using the area under the curve (AUC). We assessed model calibration with calibration curves generated by the “ResourceSelection” package. Additionally, we performed decision curve analysis (DCA) using the “decision_curve” function to evaluate the model's clinical usefulness. Finally, we conducted five-fold cross-validation using the createFolds function.

### 2.6 Subgroup analysis

To evaluate the robustness and clinical applicability of our predictive model, we performed comprehensive subgroup analyses across diverse demographic and clinical strata. Patients were stratified by sex (male vs. female) and ethnicity (Han vs. minorities). Regarding age, participants were categorized according to standard pediatric developmental stages to account for age-related physiological variations: infancy (<1 year), toddlerhood (1–3 years), preschool age (3–6 years), and school age (≥7 years). Clinical phenotypes were further differentiated by diagnostic presentation [Complete KD [CKD] vs. Incomplete KD [IKD]] and fever duration (<5 days vs. ≥ 5 days). Furthermore, we categorized patients by illness duration with specific reference to the kinetic profile of platelet count elevation. Since thrombocytosis is a hallmark of the subacute phase of KD—typically manifesting during the second week or after the first 7 days of illness—we utilized a 7-day threshold to compare patients in the early acute phase (<1 week) with those transitioning into the subacute phase (≥1 week), during which platelet counts characteristically begin to rise. The Area Under the Curve (AUC) was calculated for each subgroup to rigorously assess the model's predictive performance and consistency across these clinical contexts.

### 2.7 Statistical analysis

All statistical analyses and visualizations in our study were performed using R version 4.4.1. Normally distributed continuous variables were expressed as mean ± standard deviation (mean ± sd), and differences between groups were compared using independent samples t-tests. Non-normally distributed data were presented as medians with interquartile ranges (P25, P75), and comparisons were conducted using non-parametric rank-sum tests. Categorical variables were expressed as proportions, and group comparisons were performed using chi-square tests or Fisher's exact test. A *p*-value < 0.05 was considered statistically significant.

## 3 Results

### 3.1 Clinical characteristics description and comparison of Two groups

Our study included a total of 2,686 Kawasaki disease patients. Twelve cases met exclusion criterion (1), 27 cases met exclusion criterion (2), 589 cases met exclusion criterion (3), 433 cases met exclusion criterion (4), and 116 cases met exclusion criterion (5). A total of 1,795 patients met the diagnostic, inclusion, and exclusion criteria, with complete clinical data. Of these, 1,130 patients (62.95%) were male and 665 (37.05%) were female. Most patients were Han (1,524 cases, 84.90%), with 271 cases (15.10%) from minority ethnic groups. The mean age was 2.62 years. The IVIG-resistance group included 153 patients (8.52%), while the non-IVIG-resistance group included 1,642 patients (91.48%).

A comparison of clinical data between the IVIG-resistance and non-IVIG-resistance groups revealed significant statistical differences in various parameters, including Age, WBC, PLT, N, L, CRP, Na, APTT, TT, Fever days, Time to IVIG treatment, neutrophil/lymphocyte ratio (NLR), platelet/lymphocyte ratio (PLR), ESR, ALT, GGT, TBIL, bile acid, ethnicity (Table 1).

**Table 1:** Comparison of clinical data between the IVIG-resistance and non-IVIG-resistance groups.

Factor	Total(*n* = 1795)	IVIG-resistance (*n* = 153)	non-IVIG-resistance (*n* = 1642)	*p*
Age	2.62 ± 2.01	3.1 ± 2.13	2.58 ± 1.99	0.004
WBC	14.19 ± 5.37	19.61 ± 8.16	13.69 ± 4.72	<0.001
PLT	359.24 ± 127.04	452.48 ± 165.44	350.55 ± 119.26	<0.001
HB	112.78 ± 12.6	112.15 ± 15.05	112.84 ± 12.35	0.581
N	61.49 ± 17.38	68.62 ± 17.23	60.83 ± 17.25	<0.001
L	28.81 ± 14.82	22.47 ± 14.1	29.4 ± 14.75	<0.001
CRP	77.6 ± 50.42	108.41 ± 59.49	74.73 ± 48.52	<0.001
ALB	34.89 ± 3.74	34.28 ± 4.35	34.94 ± 3.67	0.072
GLB	26.25 ± 5.99	25.86 ± 6.44	26.29 ± 5.95	0.431
Na	135.38 ± 3.23	133.76 ± 4.49	135.53 ± 3.04	<0.001
Cl	99.5 ± 4	99.52 ± 3.95	99.49 ± 4.01	0.947
Mg	0.89 ± 0.15	0.88 ± 0.12	0.89 ± 0.15	0.392
P	1.37 ± 0.33	1.33 ± 0.34	1.38 ± 0.32	0.109
Ca	2.23 ± 0.17	2.23 ± 0.17	2.23 ± 0.17	0.738
PT	12.8 ± 3.19	13.05 ± 3.33	12.78 ± 3.18	0.331
APTT	32.64 ± 7.19	34.04 ± 7.1	32.51 ± 7.19	0.012
Fbg	5.28 ± 1.42	5.28 ± 1.24	5.28 ± 1.44	0.964
TT	16.64 ± 2.75	16.25 ± 2.33	16.68 ± 2.79	0.035
D dimer	0.51 ± 6.25	1.58 ± 15.4	0.41 ± 4.54	0.35
Fever days	5.65 ± 2.13	6.65 ± 3.14	5.55 ± 1.98	<0.001
Time to IVIG treatment	6.17 ± 1.85	6.62 ± 2.87	6.12 ± 1.72	0.037
NLR	2.38 (1.35–4.16)	3.6 (1.84–7.15)	2.3 (1.33–4)	<0.001
PLR	101.83 (69.65–147.19)	124.42 (82.21–196.96)	100.2 (68.81–142.99)	<0.001
ESR	62 (42–82)	65 (46–90)	62 (41–81)	0.025
ALT	28.7 (15–67.45)	66 (39–175)	26 (14–60)	<0.001
GGT	37 (16–94)	64.1 (34–192)	34 (15–88.75)	<0.001
TBIL	9.2 (6.5–17.4)	11.9 (8–31.4)	9.1 (6.4–15.85)	<0.001
Bile Acid	7.3 (4.8–11.4)	8.4 (5.6–16.9)	7.1 (4.8–11.2)	0.007
K	4.3 (3.8–4.8)	4.4 (3.9–4.9)	4.3 (3.8–4.8)	0.216
IgA	0.7 (0.4–0.96)	0.71 (0.4–0.99)	0.7 (0.4–0.95)	0.774
IgM	1.2 (0.84–1.42)	1.18 (0.83–1.39)	1.2 (0.84–1.44)	0.565
IgG	6.9 (4.9–9.18)	6.92 (4.91–9.18)	6.9 (4.9–9.18)	0.947
Sex				0.504
Female	665	604 (36.78)	61 (39.87)	
Male	1130	1038 (63.22)	92 (60.13)	
Ethnicity				0.003
Han ethnicity	1524113	1407 (85.69)	117 (76.47)	
Minority ethnicity	271	235 (14.31)	36 (23.53)	
Oral mucosal involvement				0.707
No	326	30 (19.61)	296 (18.03)	
Yes	1469	123 (80.39)	1346 (81.97)	
Conjunctival injection				0.531
No	247	18 (11.76)	229 (13.95)	
Yes	1548	135 (88.24)	1413 (86.05)	
Rash				0.327
No	501	37 (24.18)	464 (28.26)	
Yes	1294	116 (75.82)	1178 (71.74)	
Cervical lymphadenopathy				0.686
No	946	75 (49.02)	771 (46.95)	
Yes	949	78 (50.98)	871 (53.05)	
Symptoms of limb				0.646
No	936	83(54.25)	853(51.95)	
Yes	859	70(45.75)	789(48.05)	

### 3.2 Screening for Key factors

**Figure 1:**
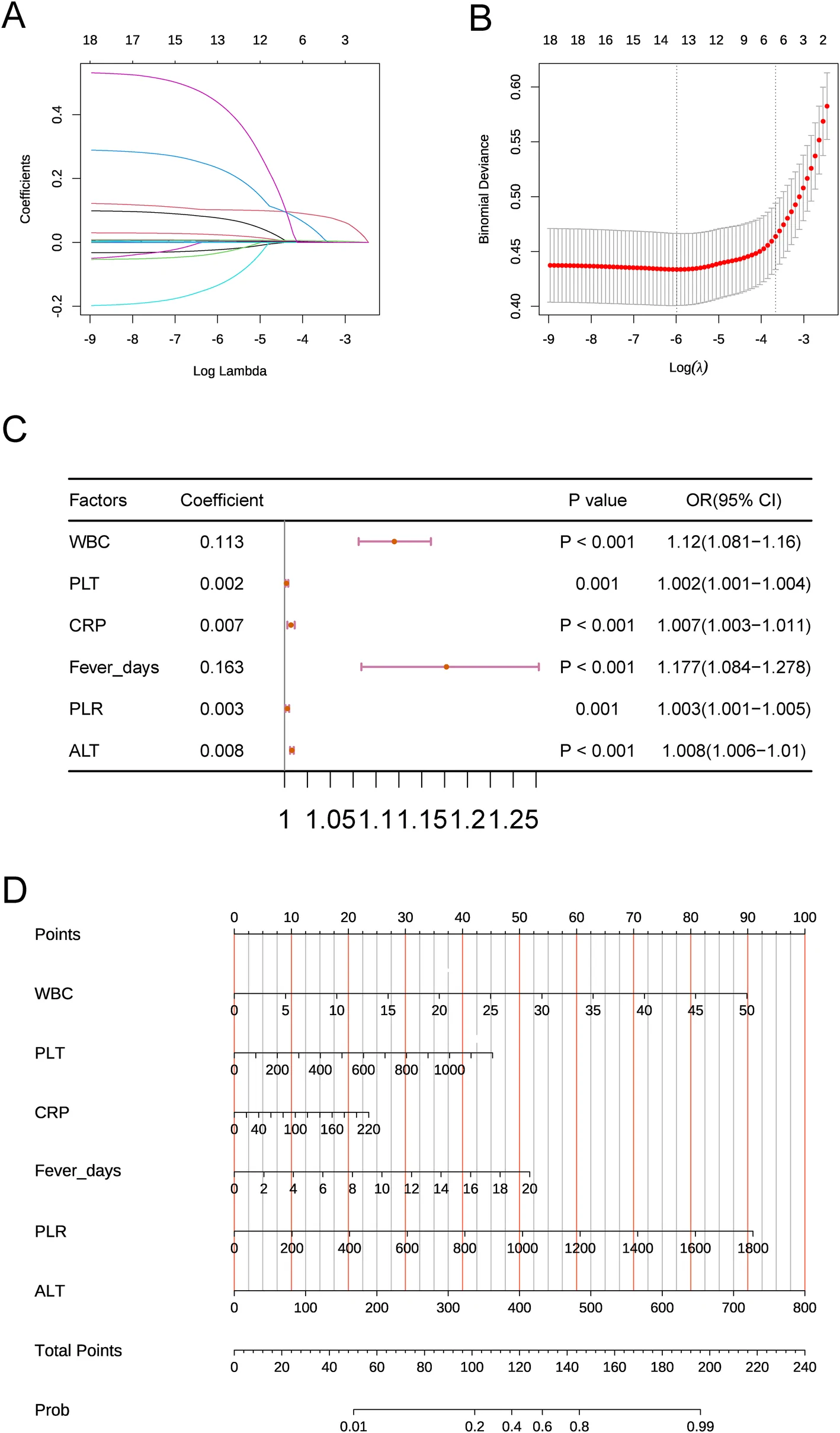
**A,B**, Key variables selected by LASSO. **C**, Forest plot of the multivariable logistic regression model. **D**, Nomogram of the risk model.

To simplify the risk model, we included the 19 factors in a LASSO analysis to identify the most predictive variables. We used 10-fold cross-validation to select the optimal *λ* value of 0.0257. A higher *λ* value increased regularization, reducing the number of selected variables. Ultimately, we selected WBC, PLT, CRP, fever days, PLR, and ALT (Figures 1A,B). These six factors were incorporated into the subsequent model.

To address multicollinearity, we performed correlation analysis on the six key factors. Pearson correlation coefficients were all below 0.5, with a maximum of 0.34. This indicated a low risk of multicollinearity bias.

### 3.3 Construction and evaluation of the risk model

We also analyzed the impact of six key factors on IVIG resistance in KD. After including them in the multivariate logistic regression model, higher WBC, PLT, CRP, fever days, PLR, and ALT were identified as independent risk factors for IVIG resistance (Figure 1C). We further constructed a nomogram to visualize the risk model (Figure 1D). We calculated the Youden index of predictive model to be 0.505, and based on this, determined the cutoff value for the risk score to be 5.701. A prognostic risk score was constructed based on the following formula:

$$\begin{array}{rl} & [\mathrm{Risk}\;\mathrm{score}=0.113*\mathrm{WBC}+\mathrm{PLT}*0.002+\mathrm{CRP}*0.007\\ & \;+\;\mathrm{Fever}\;\mathrm{days}*0.163+\mathrm{PLR}*0.003+\mathrm{ALT}*0.008] \end{array}$$

**Figure 2:**
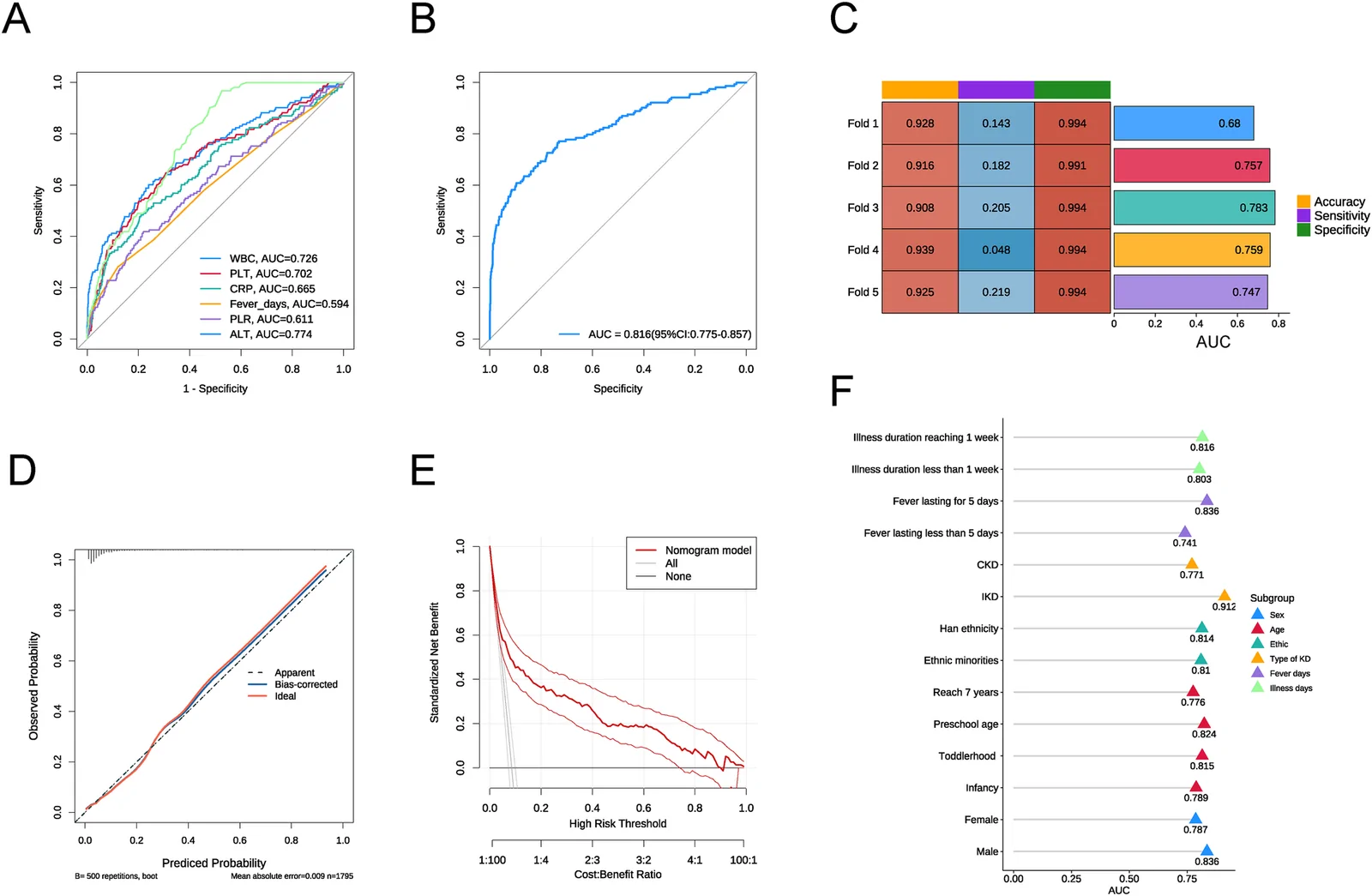
**A**, ROC curve of the risk model; **B**, ROC curves for individual factors: WBC, PLT, CRP, fever days, PLR, and ALT; **C**, results of the 5-fold cross-validation; **D**, calibration curve of the risk model; **E**, DCA curve of the risk model; **F**, AUC values for each subgroup.

To compare the predictive performance between the model and single risk factors, we first plotted ROC curves for six independent risk factors, with ALT achieving the highest AUC of 0.774 (Figure 2A). Next, we plotted the ROC curve for the multivariate logistic regression model, which had an AUC of 0.816 (95% CI: 0.775–0.857), accuracy of 0.931, sensitivity of 0.275, and specificity of 0.992 (Figure 2B). The calibration curve demonstrated good consistency of the risk prediction model (Figure 2C). DCA further confirmed its accuracy (Figure 2D). We performed 5-fold cross-validation to validate the model's predictive performance, showing it achieved good efficacy (Figure 2E).

### 3.4 Subgroup analysis

To evaluate our model's predictive performance under different conditions, we conducted subgroup analyses based on sex, age, ethnicity, KD type, fever days, and illness duration. The model demonstrated good predictive value in each subgroup (Figure 2F). The AUC for fever lasting 1 week was 0.816, higher than the 0.803 for less than 1 week. The AUC for KD with 5 days of fever was 0.836, higher than 0.741 for less than 5 days. The AUC for IKD was 0.912, significantly higher than 0.771 for CKD. The AUC for Han ethnicity was 0.814, slightly higher than 0.810 for minorities. Males had an AUC of 0.836, higher than 0.787 for females. In different pediatric stages, the AUC was 0.789 for infants, 0.815 for toddlers, 0.824 for preschoolers, and 0.776 for children reaching 7 years.

## 4 Discussion

IVIG combined with aspirin is the recommended first-line treatment for the acute phase of KD. Some children exhibit IVIG resistance, which increases CAL incidence, extends hospitalization, and raises disease burden (11–13). We conducted a retrospective analysis of 1,795 KD patients’ demographic, clinical, and laboratory features. We developed a multivariate logistic regression model, used LASSO analysis for variable selection, and performed five-fold cross-validation. We also conducted subgroup validation based on different clinical features. Unlike previous studies, our research included immune-related markers, electrolytes, and multiple clinical characteristics, rather than only early inflammatory markers and routine biochemistry. Additionally, we used a larger KD cohort for model development. We addressed heterogeneity between clinical features and outcomes through subgroup validation.

KD is an acute immune-mediated vasculitis primarily affecting children under five years of age. In developed countries, KD has surpassed acute rheumatic fever to become the leading cause of acquired heart disease (1). The most severe complication is the development of CAL, accounting for nearly all KD-related deaths. The use of IVIG effectively reduces vascular inflammation and decreases the incidence of CAL from approximately 25% to 5%–10%. However, about 10%–20% of patients exhibit resistance to IVIG, which increases the risk of CAL (14–16). The resistance may be related to the dynamic activation state of macrophages and serum cytokine levels during acute KD. Improper timing of IVIG administration can disturb the balance between pro-inflammatory and anti-inflammatory cytokines, impacting clinical outcomes, including the need for additional treatments and the development of CAL. Previous studies have shown that IVIG influences the immune regulation involving dual TCR T cells, particularly CD8+ T cells and Treg cells (17). Unfortunately, the mechanisms underlying IVIG resistance in KD remain poorly understood, and specific pathogenic pathways in resistant cases have yet to be elucidated. Since TCR T cells are crucial for the immune response to IVIG in KD, future research will focus on further investigating TCR T cell-related immune responses in patients resistant to IVIG. Apart from Japan's Egami, Kobayashi, and Sano scores (18, 19), and Taiwan's Formosa score (7, 20), several predictive models for IVIG resistance have been developed in various regions within China, such as Beijing, Fujian and Suzhou (21, 22). However, the reported results of these models appear to be somewhat inconsistent. Consequently, the external validity of most existing prediction models for IVIG resistance in KD may remain, to some extent, uncertain. The predictive performance of locally developed scores could vary considerably, with sensitivities ranging from 0.486 to 0.811 and specificities from 0.366 to 0.816. This observation potentially aligns with current evidence suggesting that the predictive effectiveness of these scores might exhibit notable regional specificity, possibly due to geographic or ethnic variations (21–24).

Our study found that higher WBC, PLT, CRP, fever days, PLR, and ALT were independent risk factors for IVIG resistance in KD. Both WBC and CRP indicated a stronger inflammatory response. Elevated WBC reflected an intense immune reaction to inflammation, suggesting a high level of immune activation. CRP, as an acute-phase protein, was produced by hepatocytes stimulated by pro-inflammatory cytokines such as IL-6, IL-1β, and TNF-α. Elevated WBC and CRP reflected more severe inflammation. Elevated levels of WBC and CRP are frequently associated with the occurrence of cytokine storms (characterized by mediators such as IL-6, TNF-α, and IL-1β). The persistent elevation of these cytokines might contribute to the continuous activation of canonical inflammatory pathways, including NF-*κ*B and JAK/STAT. Such a sustained pro-inflammatory environment appears to, at least potentially, pose a challenge to the rapid immunomodulatory effects of IVIG, possibly hindering its ability to promptly suppress the systemic immune response (25). As a result, the anti-inflammatory and immunomodulatory effects of IVIG became limited, leading to treatment resistance. KD caused endothelial injury in small and medium blood vessels and inflammatory cytokine stimulation, thereby activating platelets and promoting thrombocytosis and platelet aggregation. Increased PLT and PLR indicated platelet-driven pro-inflammatory and vascular injury mechanismsIt is widely recognized that KD is characterized by endothelial injury in small and medium blood vessels, which, coupled with inflammatory cytokine stimulation, may trigger platelet activation, thrombocytosis, and enhanced aggregation. Elevated PLT and PLR could, therefore, serve as potential surrogates for platelet-driven pro-inflammatory pathways and vascular injury mechanisms (26). Activated platelets appear to release a diverse array of inflammatory mediators that might further exacerbate endothelial damage, potentially establishing a self-perpetuating ‘vicious cycle.’ Notably, a high PLR may indirectly reflect an imbalance where the pro-inflammatory response—driven by platelet-derived cytokines—potentially overwhelms immunosuppressive mechanisms, such as those involving the regulatory molecules CTLA-4 and PD-1 on lymphocytes. Furthermore, it has been hypothesized that an increased platelet count might provide an expanded pool of Fc*γ*R receptors. These receptors could potentially compete for IVIG binding, thereby conceivably attenuating the overall immunomodulatory efficacy of IVIG treatment (27). Longer fever duration reflected prolonged inflammation. Persistent fever indicated that the immune system could not self-regulate against pro-inflammatory stimuli, leading to more severe and sustained inflammation. Prolonged inflammatory and immune cell activation impaired immune regulatory mechanisms, such as reduced Treg suppressive function, which increased the threshold of the inflammatory cytokine network and reduced the effectiveness of IVIG modulation. Elevated ALT indicated liver inflammation and parenchymal injury. As systemic inflammation worsened, hepatic tissues in KD patients became more susceptible to injury, resulting in hepatocyte necrosis or dysfunction. At this stage, inflammation was not limited to peripheral blood and vessels but also affected the liver, indicating a continuous elevation of pro-inflammatory cytokines (IL-6, TNF-α, IL-1β, etc.) and high immune activation (28). This led to declines in acute phase proteins and immunomodulatory molecules synthesized by the liver, such as CRP, complement, and scavenger receptors, disrupting the clearance of inflammation and immune homeostasis (29). Moreover, liver injury promoted persistently high levels of pro-inflammatory cytokines, suppressed the synthesis of anti-inflammatory factors, and broke the inflammatory balance, thereby aggravating IVIG resistance. Hepatic dysfunction might also delay IVIG metabolism, reducing its efficiency in immunoregulation and anti-inflammation.

In our study, we observed substantial differences in predictive performance among subgroups. The prediction accuracy for the IKD subgroup was significantly higher than for the CKD subgroup. Currently, KD diagnosis mainly relied on the duration of fever and typical clinical features. IKD was often diagnosed and treated later due to atypical clinical presentations. As a result, laboratory indicators such as WBC, CRP, PLT, PLR, and ALT were elevated in IKD, reflecting more severe, prolonged inflammation and organ damage. Therefore, the prediction model showed higher efficacy in this subgroup. Conversely, early diagnosis and intervention in CKD reduced the association between laboratory markers and IVIG resistance, resulting in lower predictive accuracy.

This study integrated inflammation markers (CRP, WBC), immune-metabolic indicators (PLT, PLR), liver function parameters (ALT), and fever duration. It provided a robust foundation for developing a standardized clinical prediction model. However, the retrospective, single-center design may have introduced recall bias. The lack of external validation across different regions, ethnicities, and time periods limited the generalizability of our findings. Consequently, the applicability of the model in broader clinical settings remained restricted. Future efforts should include multicenter, prospective studies with large samples and diverse populations to enhance the model's reliability and practical value.

## 5 Conclusions

In conclusion, our model demonstrated strong predictive ability for IVIG resistance in KD patients and was validated across various factors, including age, sex, ethnicity, KD type, duration of fever, and disease course. This model aimed to provide supplementary decision-making support for frontline treatment of KD, assisting in the early identification of IVIG resistance and enabling timely interventions to protect the cardiovascular health of KD patients.

## Data Availability

The raw data supporting the conclusions of this article will be made available by the authors, without undue reservation.
